# ARE OLDER ADULTS ABLE TO DRAW UPON PRIOR EXPERIENCES WHEN COPING WITH THE NOVEL COVID-19 STRESSOR?

**DOI:** 10.1093/geroni/igac059.1957

**Published:** 2022-12-20

**Authors:** Maria Kurth, Heidi Igarashi, Hye Soo Lee, Soyoung Choun, Carolyn Aldwin

**Affiliations:** Oregon State University, Corvallis, Oregon, United States; Oregon State University, Corvallis, Oregon, United States; Oregon State University, Corvallis, Oregon, United States; Oregon State University, Corvallis, Oregon, United States; Oregon State University, Corvallis, Oregon, United States

## Abstract

Despite higher physiological vulnerability to stress, older adults may accumulate resources through prior experiences that can promote resilience (Aldwin & Igarashi, 2016). During the COVID-19 pandemic, older adults drew on prior experiences and resources to cope (McKinlay et al., 2021; Herron et al., 2021), although these events were typically not specified. Some found vulnerability due to prior trauma (Galica et al., 2021). We examined whether older adults drew upon specific experiences or more general resilience resources in coping with this novel stressor. Data were collected using an online survey from April 28-May 4, 2020 from 235 older adults in Oregon (Mage = 71.35, SD = 7.39; 74% female; 92% White). We examined open-ended responses from a question that asked whether prior experiences influenced how they were dealing with the COVID-19 situation. Nearly 2/3 provided valid responses (n=144). After inductive open coding, preliminary consolidation resulted in three broad categories: past experiences (74%), resources (19%), and both (8%). The most common prior experiences were illness (n = 20) and work (n = 19). Some (n=10) reported specific coping strategies learned during prior stressful experiences. Resources include personal characteristics (e.g., being “introverted” or “resilient”), financial (“financially secure”) and social resources (“loving spouse”). Five reported experiences that made COVID-19 more difficult (“PTSD/anxiety prior to COVID-19 makes this even worse”). Although 1/3 of the sample could not draw upon a prior experience in coping with this novel stressor, many older participants could utilize their lived experience when coping with problems during the COVID-19 pandemic.

